# The role of vitamin D in sleep regulation: mechanisms, clinical advances, and future directions

**DOI:** 10.3389/fnut.2025.1595813

**Published:** 2025-06-02

**Authors:** Zhilong Cai, Shuoyu Rui, Nanqu Huang, Fei Feng, Yong Luo

**Affiliations:** ^1^Department of Neurology, Third Affiliated Hospital of Zunyi Medical University (The First People’s Hospital of Zunyi), Zunyi, Guizhou, China; ^2^National Drug Clinical Trial Institution, Third Affiliated Hospital of Zunyi Medical University (The First People’s Hospital of Zunyi), Zunyi, Guizhou, China

**Keywords:** vitamin D, sleep regulation, vitamin D receptor, sleep disorders, single-cell sequencing

## Abstract

Vitamin D, an essential neuroendocrine regulator, exhibits a significant dose-dependent association with various sleep disorders according to epidemiological evidence, and plays a multifaceted and critical role in sleep regulation. This review summarizes the molecular mechanisms, clinical applications, and future directions of vitamin D in sleep regulation. Vitamin D may influence sleep through multiple molecular pathways, including modulation of neurotransmitter systems, maintenance of circadian rhythms, and neuroimmune regulation. Clinical trials have demonstrated that vitamin D supplementation significantly improves sleep quality, particularly in special populations such as the elderly and pregnant women. However, challenges persist in optimizing individualized treatment regimens, developing novel drug delivery systems, and evaluating long-term efficacy. The integration of multi-omics analyses and artificial intelligence offers innovative solutions to these challenges. Future research should focus on elucidating the precise molecular mechanisms of vitamin D in sleep regulation, refining personalized therapeutic strategies, and advancing innovative delivery systems to enhance the prevention and treatment of sleep disorders.

## 1 Introduction

Vitamin D plays a multifaceted role in sleep regulation, and its relationship with sleep disorders has garnered increasing scientific attention. Recent studies have elucidated multiple mechanisms through which vitamin D participates in sleep modulation processes and its emerging significance in the pathogenesis, progression, and therapeutic management of sleep disorders. As a prevalent condition severely impacting physical and mental health, sleep disorders encompass seven major categories according to the International Classification of Sleep Disorders-Third Edition (ICSD-3), including insomnia disorders, sleep-related breathing disorders, and central disorders of circadian rhythm (1). Epidemiological studies indicate that approximately 30% of the global population experiences varying degrees of sleep disturbances, with notable disparities in chronic insomnia prevalence across different demographics (2). These disorders not only substantially impair quality of life but also demonstrate significant associations with the development and progression of chronic conditions including cognitive dysfunction, cardiovascular diseases, diabetes mellitus, and depression, while concurrently elevating risks of accidental injuries (3, 4). Although current therapeutic strategies such as pharmacological treatments and cognitive behavioral therapy (CBT) remain mainstays of clinical management, limitations including drug dependency risks and prolonged treatment duration (5), underscore the critical need for novel therapeutic targets and intervention strategies.

In recent years, vitamin D has garnered increasing attention as a critical neuroendocrine regulator in sleep modulation. Studies have demonstrated a significant association between vitamin D deficiency and various sleep disorders (6, 7),with serum vitamin D levels showing positive correlations with sleep quality, duration, and efficiency metrics (8). Notably, vitamin D supplementation has been shown to markedly improve sleep quality and depressive symptoms in elderly populations (9). Furthermore, clinical investigations in chronic pain patients revealed that vitamin D intervention significantly enhances both sleep parameters and quality of life (10). At the mechanistic level, vitamin D exerts its regulatory effects through widespread expression of its receptor (VDR) in key brain regions, profoundly influencing complex physiological processes underlying sleep regulation (11, 12). The proposed mechanisms encompass modulation of neurotransmitter metabolism, maintenance of neural plasticity, regulation of inflammatory cytokines and oxidative stress markers, and potential interactions with melatonin secretion pathways (13–15). Molecular studies elucidate that vitamin D regulates core circadian rhythm genes (e. g, Clock, Bmal1, and Per2) through VDR-mediated signaling pathways, while concurrently modulating expression of brain-derived neurotrophic factor (BDNF), a neurotrophin critically involved in sleep homeostasis (16–20). Emerging research has further unveiled a novel mechanism whereby vitamin D deficiency may disrupt sleep regulation through impaired neuroglial cell functionality (21). Despite these advances, critical challenges persist in translating vitamin D research into clinical sleep medicine, including the need for individualized supplementation regimens, long-term efficacy evaluation, causal relationship verification, and elucidation of synergistic interactions with other sleep-regulatory factors. Addressing these knowledge gaps represents a crucial direction for future research in this evolving field.

Therefore, this review aims to systematically synthesize current evidence on the mechanistic roles, clinical applications, and critical challenges of vitamin D in sleep–wake regulation. Specifically, we will focus on: (1) Molecular mechanisms underlying vitamin D-mediated sleep modulation; (2) Clinical evidence supporting vitamin D intervention efficacy; (3) Population-specific associations between vitamin D status and sleep quality parameters; (4) Optimization of personalized therapeutic strategies. Through comprehensive analysis of existing evidence, this review will identify critical knowledge gaps and propose innovative research directions, ultimately advancing evidence-based clinical decision-making in sleep medicine.

## 2 Molecular mechanisms of vitamin D in sleep regulation

### 2.1 Distribution patterns of vitamin D receptor in sleep-related brain regions

The VDR exhibits a complex and region-specific distribution across sleep-regulatory brain nuclei, with prominent expression observed in the suprachiasmatic nucleus (SCN) of the hypothalamus (18, 19). As the central circadian pacemaker in mammals, the SCN orchestrates precise regulation of sleep–wake cycles through intricate neural networks while maintaining functional connectivity with olfactory systems to coordinate circadian outputs (20). At the neuronal subpopulation level, VDR in the SCN localizes to two critical neuron subtypes: vasoactive intestinal peptide (VIP)-positive and arginine vasopressin (AVP)-positive neurons. VIP neurons primarily mediate photic entrainment and intra-SCN synchronization, whereas AVP neurons play pivotal roles in determining circadian periodicity (22, 23). Emerging evidence suggests this differential expression correlates with Brain and Muscle ARNT-Like 1 (BMAL1) expression levels, a core clock gene directly influencing circadian oscillator robustness (24). Molecular analyses reveal VDR interacts with retinohypothalamic tract (RHT) signaling pathways to modulate light input transduction to the SCN. Circadian regulation involves transcriptional-translational negative feedback loops of clock genes (25), which govern physiological processes including sleep–wake cycles, body temperature rhythms, endocrine activity, and metabolic homeostasis. Notably, in Agouti-related peptide (AgRP) neurons, this regulation extends to circadian control of metabolism and hunger signaling (26, 27). Photic signals transmitted via the RHT activate specific SCN neuronal subpopulations, triggering expression of core clock genes. VDR coordinates with VIP/AVP signaling to regulate this molecular network through transcriptional and post-transcriptional mechanisms, ensuring precise circadian synchronization. Intriguingly, BMAL1 exhibits dual functionality, not only governing circadian rhythms but also influencing cell cycle progression via telomeric repeat-containing RNA (TERRA) regulation and telomeric heterochromatin organization (28). Recent studies further identify functional interplay between VDR and Period2 (Per2) in neuroglial cells, significantly impacting emotion-related behavioral regulation (29, 30). This discovery provides novel insights into VDR’s role within sleep-emotion regulatory networks, highlighting its multidimensional contributions to neurocircuitry integration.

### 2.2 Vitamin D modulation of neurotransmitter systems

Vitamin D exerts multifaceted regulatory effects on neurotransmitter systems critical for sleep–wake cycle regulation through distinct molecular pathways. Emerging evidence highlights a bidirectional interaction between vitamin D and melatonin biosynthesis systems (21). The VDR directly regulates transcriptional activity of arylalkylamine N-acetyltransferase (AANAT), a rate-limiting enzyme in melatonin synthesis (31). Under chronic stress conditions, melatonin dysfunction-associated sleep disturbances may arise from vitamin D-mediated epigenetic regulation of AANAT and acetylserotonin O-methyltransferase (ASMT) gene expression (32, 33). Within the serotonergic system, vitamin D plays a pivotal role in serotonin synthesis by modulating the expression of tryptophan hydroxylase 2 (TPH2) and the activity of serotonin transporters. This process may interact with histaminergic receptor system regulation, and structural variations in TPH2 could influence the efficiency of vitamin D-mediated serotonin synthesis (34). Notably, vitamin D also affects GABAergic neuronal function through epigenetic mechanisms, specifically regulating the expression of glutamic acid decarboxylase (GAD) and histone H3 acetylation, particularly under stress conditions (35). Recent studies further reveal that vitamin D regulates the development of GABAergic neurons during neural stem cell differentiation (36). Importantly, this regulatory effect exhibits significant neuron subtype specificity, with distinct responsiveness to vitamin D observed across different GABAergic neuron subtypes.

### 2.3 Vitamin D-mediated neuroimmune regulation

Vitamin D participates in neuroimmune regulation through multiple mechanisms, playing a pivotal role in neuroprotection and sleep modulation. The impact of gut microbiota (GM) derived metabolites—such as short-chain fatty acids (SCFAs) and tryptophan catabolites—on the neuroimmune system and hypothalamic pituitary adrenal (HPA) axis has been well described (37), further supporting their role in the pathophysiology of anxiety and depression. Studies demonstrate that the VDR modulates neuroinflammatory responses by suppressing the NF-κB signaling pathway, thereby reducing pro-inflammatory cytokine expression (e.g., TNF-*α*, IL-1β, and IL-6) in patients with chronic stress-induced sleep disturbances (38, 39). This anti-inflammatory action effectively mitigates sleep disorders associated with melatonin dysfunction. Furthermore, vitamin D exerts neuroprotective effects by regulating brain-derived neurotrophic factor (BDNF) expression, influencing sleep–wake cycle regulation and cognitive enhancement. For instance, vitamin D improves cognitive function and sleep quality through modulation of pro-brain-derived neurotrophic factor (proBDNF) expression levels in the hippocampal region (40). Vitamin D also enhances blood–brain barrier (BBB) integrity by upregulating tight junction proteins (e.g., claudin-5 and occludin), a critical mechanism for maintaining normal sleep–wake rhythms (41). Under inflammatory conditions induced by chronic sleep deprivation, vitamin D protects tight junction proteins from degradation by inhibiting matrix metalloproteinase (MMP) activity (42).

## 3 Clinical advancements in vitamin D intervention

### 3.1 Association between vitamin D and sleep quality

Recent epidemiological studies have revealed a significant dose-dependent relationship between vitamin D status and sleep quality (43). In dialysis patients, improvements in vitamin D levels are associated with marked enhancements in sleep quality (44). However, this association exhibits age- and sex-dependent heterogeneity in the general population. For instance, elevated vitamin D levels correlate with improved sleep quality in middle-aged populations (CAT score: −0. 35, 95% CI: −0. 67 to −0. 03, *p* = 0. 03), while no significant association is observed in older adults aged ≥65 years, suggesting age as a critical moderating factor (45). Sex-specific analyses demonstrate that higher serum vitamin D levels inversely associate with cognitive/affective symptoms in males (IRR = 0. 56, 95% CI: 0. 40–0. 80), whereas altered sleep patterns in pregnant women show strong correlations with vitamin D status (46). These sex disparities may arise from hormonal regulation of vitamin D metabolism. Objective sleep parameter analyses further corroborate this relationship. Actigraphy-based studies reveal significant associations between serum vitamin D concentrations and multiple sleep metrics (47). Individuals with vitamin D insufficiency (<20 ng/mL) exhibit prolonged sleep latency and reduced sleep efficiency, particularly among older adults, where deficiency correlates with circadian rhythm disruptions and cognitive decline (48). These findings underscore vitamin D’s multifaceted mechanistic involvement in sleep quality regulation, warranting deeper investigation into its therapeutic potential.

### 3.2 Clinical trials of vitamin D supplementation

Recent advancements in clinical trials have highlighted the therapeutic potential of vitamin D supplementation in improving sleep quality and overall health. Evidence indicates vitamin D supplementation not only improves sleep quality through multiple mechanisms but also significantly alleviates depressive symptoms and optimizes sleep metrics in perinatal women (49). Furthermore, high-dose vitamin D supplementation (>4,000 IU/day) has shown superior efficacy over standard doses in reducing sleep latency and enhancing sleep efficiency, though its long-term safety requires further validation (50). Emerging evidence supports personalized supplementation protocols tailored to patients’ baseline vitamin D status and clinical profiles. Synergistic effects have been observed in combinatorial nutrient interventions, such as co-administration with melatonin, which amplifies sleep quality improvements (51). Combined magnesium and vitamin D supplementation not only enhances sleep outcomes but may also benefit mental health in individuals with attention-deficit/hyperactivity disorder (ADHD). Population-specific studies reveal novel insights: vitamin D supplementation in pregnant women improves both sleep quality and pregnancy outcomes (52), while shift workers exhibit enhanced circadian rhythm alignment and sleep consolidation (53) Chronic disease populations further demonstrate quality-of-life improvements correlated with vitamin D intervention (54), These findings inform novel directions for personalized therapeutics and combination strategies in clinical practice. However, the lack of long-term follow-up data precludes comprehensive evaluation of intervention efficacy, while population-specific applicability remains underexplored. Addressing these limitations will require large-scale, multicenter randomized controlled trials with stratified participant cohorts and mechanistic sub-studies.

### 3.3 Clinical applications in special populations

Vitamin D supplementation demonstrates distinct clinical benefits across special populations, with emerging research underscoring the critical importance of personalized interventions tailored to individual characteristics. In older adults, vitamin D supplementation not only improves sleep quality but also significantly alleviates depressive symptoms (55), while exerting positive effects on mental health and sleep parameters, though individualized dose optimization remains essential (56). Among psychiatric patients, vitamin D supplementation may ameliorate depressive and anxiety symptoms through modulation of neurotransmitter systems and inflammatory markers (57), showing particular promise for improving outcomes in early-stage psychosis (58). In young adults with depression, vitamin D status correlates significantly with impulsivity traits and personality dimensions (59). Recent evidence suggests that gut microbiota alterations are associated with mood symptoms even in adolescent populations, emphasizing the potential of early microbiome-based interventions (60). Notably, hospitalized pediatric patients exhibit dual benefits from supplementation, including enhanced sleep quality and reduced hospitalization duration (61). Longitudinal follow-up studies further suggest that early-life vitamin D interventions may confer lasting mental health advantages during later childhood (62). In pregnant populations, vitamin D supplementation demonstrates multifaceted value by improving sleep quality, optimizing pregnancy outcomes, and reducing risks of adverse gestational complications. These findings collectively highlight the broad clinical potential of precision vitamin D interventions across diverse special populations.

## 4 Optimization strategies and future perspectives for vitamin D intervention

### 4.1 Development of personalized therapeutic regimens

Personalized vitamin D intervention has emerged as a significant advancement in precision medicine, propelled by machine learning (ML) and computational techniques. These approaches have transcended traditional protocol limitations by facilitating dynamic supplementation models tailored to individual needs (63). Advanced algorithms, including support vector machines, neural networks, and ensemble methods such as extreme gradient boosting (XGBoost) and random forests, effectively integrate multi-omics data (e.g., genomic, metabolomic) to predict vitamin D status with remarkable precision (64–67). Hybrid models incorporating Bayesian optimization have successfully identified key predictive features, enabling the development of adaptive protocols that automatically adjust supplementation regimens based on individual characteristics (68–70). Notably, integrated prediction models combining clinical and biomarker data have demonstrated exceptional performance (AUC = 0. 920) and robust external validity across diverse populations, establishing reliable decision-support frameworks for clinical implementation (71, 72).

### 4.2 Development of novel delivery systems

Recent innovations in drug delivery technologies have revolutionized vitamin D administration, addressing longstanding challenges of poor bioavailability and stability (73). Advanced carrier systems, including polysaccharide-based nanogels, mesoporous silica platforms, and lipid-based nanovehicles, have significantly enhanced vitamin D solubility and absorption profiles (74–76). These sophisticated delivery mechanisms incorporate stimuli-responsive features that enable precise spatiotemporal control over drug release, substantially improving targeting specificity to affected tissues (77). Through meticulous engineering of carrier material architectures and physicochemical properties, researchers have substantially improved vitamin D stability and *in vivo* absorption kinetics, laying critical technological foundations for advancing personalized and precision medicine (78–80). Furthermore, biomaterial-based delivery systems exhibit exceptional biocompatibility while enhancing drug absorption through multimodal mechanisms (81). These technological breakthroughs represent a significant advancement in therapeutic approaches, establishing robust foundations for personalized medicine applications and more effective management of vitamin D-related conditions.

### 4.3 Future research directions

Vitamin D research currently confronts significant scientific challenges, including cellular response heterogeneity and analytical limitations that impede comprehensive understanding of its biological functions (82–84). Divergent cellular reactions to vitamin D substantially complicate mechanistic investigations, while existing methodologies face bottlenecks in sample processing and data analysis (85). To overcome these obstacles, researchers are increasingly adopting advanced single-cell sequencing technologies that effectively resolve cellular heterogeneity and enable dynamic monitoring of gene expression profiles (86–88). These approaches reveal crucial insights into vitamin D’s molecular regulatory networks, with single-cell transcriptomics identifying key signaling pathways involved in processes such as sleep modulation (89). Furthermore, multi-omics integration expands research capabilities by systematically examining vitamin D’s biological roles across complementary data dimensions, advancing the field toward more nuanced mechanistic understanding (90).

Clinical translation faces dual challenges: developing personalized regimens and evaluating therapeutic efficacy. Research demonstrates significant interindividual variability in responses to vitamin D interventions (91), driving precision medicine advancements through machine learning-driven predictive models and innovative drug delivery systems (92, 93). Emerging detection technologies hold promise for identifying novel biomarkers and refining therapeutic strategies.

Future research should prioritize three key directions: validating vitamin D’s sleep-regulatory mechanisms through rigorous clinical trials; developing personalized dosing protocols with advanced delivery platforms; and fostering innovation through interdisciplinary technology integration. Simultaneously, establishing ethical guidelines and conducting cost-effectiveness analyses should guide translational efforts from a global health perspective. This coordinated approach will help unlock vitamin D’s full therapeutic potential, delivering precise health solutions for diverse populations worldwide.

## 5 Conclusion

**Figure 1:**
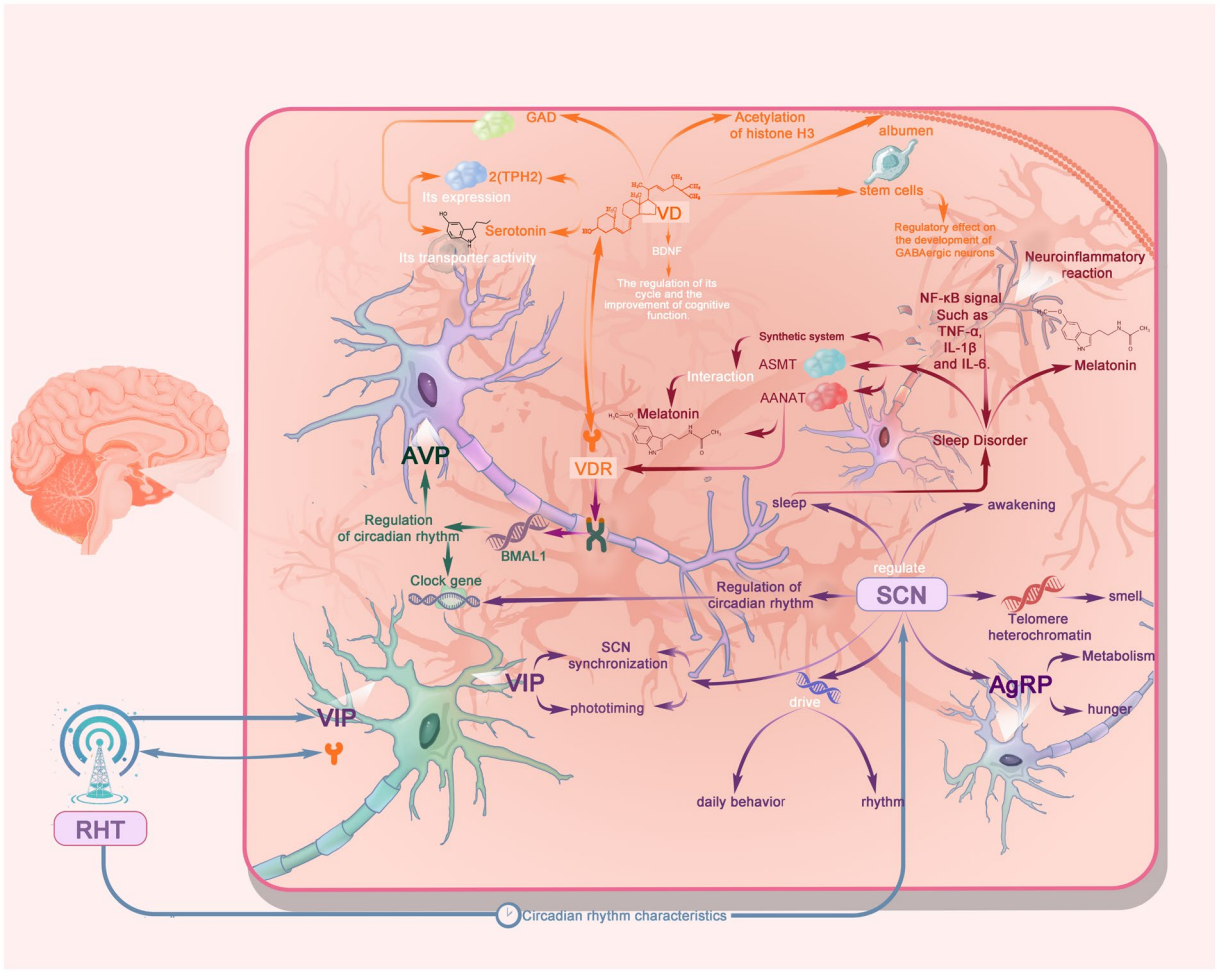
Simplified schematic of the molecular mechanisms of vitamin D (VD) in sleep regulation. In the diagram, VD exerts multiple effects by binding to the vitamin D receptor (VDR).

Vitamin D plays a critical role in sleep regulation, with significant advancements achieved in elucidating its molecular mechanisms (Figure 1) and clinical applications. The integration of cutting-edge technologies such as single-cell sequencing has provided novel insights into the spatial distribution and functional significance of vitamin D receptors (VDRs) within sleep-related brain regions. Artificial intelligence approaches, including machine learning, have substantially enhanced the precision of personalized therapeutic strategies, offering robust support for clinical decision-making. However, persistent challenges remain, including the complexity introduced by cellular heterogeneity, limitations in current analytical methodologies, and insufficient long-term follow-up data. Future research should focus on: 1) in-depth exploration of the detailed molecular mechanisms by which vitamin D regulates sleep; 2) optimization of personalized therapeutic strategies; 3) clinical translation of novel drug delivery systems; and 4) enhancement of multicenter clinical trials. Through interdisciplinary integration and the application of new technologies, research on vitamin D in the field of sleep medicine will continue to achieve breakthrough progress, providing more effective solutions for the prevention and treatment of sleep disorders.
